# Identification of a Protein with Antioxidant Activity that is Important for the Protection against Beer Ageing

**DOI:** 10.3390/ijms12096089

**Published:** 2011-09-19

**Authors:** Ming J. Wu, Frank M. Clarke, Peter J. Rogers, Paul Young, Narelle Sales, Patrick J. O’Doherty, Vincent J. Higgins

**Affiliations:** 1 School of Biomedical and Health Sciences, College of Health and Science, University of Western Sydney, Locked Bag 1797, Penrith South DC, New South Wales 1797, Australia; E-Mail: p.odoherty@uws.edu.au; 2 School of Science, Griffith University, Nathan, Queensland 4111, Australia; E-Mail: f.clarke@griffith.edu.au; 3 Carlton and United Breweries, Fosters Group, 4-6 Southampton Crescent, Abbotsford, Melbourne 3067, Australia; E-Mail: peterjohnrogers@me.com; 4 Department of Primary Industry NSW, Elizabeth Macarthur Agricultural Institute, PMB 8, Camden, New South Wales 2570, Australia; E-Mails: paul.young@industry.nsw.gov.au (P.Y.); narelle.sales@industry.nsw.gov.au (N.S.); 5 Ramaciotti Centre for Gene Function Analysis, School of Biotechnology and Biomolecular Sciences, University of New South Wales, NSW 2052, Australia

**Keywords:** beer thiol protein, flavour, free radical, antioxidant, LTP1, yeast, *Saccharomyces cerevisiae*

## Abstract

This study was carried out with fresh Australian lager beer which was sampled directly off the production line, the same samples aged for 12 weeks at 30 °C, and the vintage beer which was kept at 20 °C for 5 years. Characteristic Australian lager flavour was maintained in the fresh and vintage beers but was lost in the aged beer. Sodium dodecyl sulphate polyacrylamide gel electrophoresis (SDS-PAGE) and free thiol group labelling analyses of beer proteins found that this flavour stability correlated with the presence of an unknown 10 kilodaltons (kDa) protein with a higher level of free thiols. The protein was purified by size-exclusion chromatography, then peptide sequencing and database matching identified it as the barley lipid transfer protein (LTP1). Further characterisation using diphenylpicrylhydrazyl (DPPH) free radical scavenging and a *Saccharomyces cerevisiae*-based antioxidant screening assay demonstrated that the LTP1 protein was active in DPPH reduction and antioxidant activity. The absence of free thiol in the aged beer indicates that the thiol functional groups within the LTP1 protein were saturated and suggests that it is important in the flavour stability of beer by maintaining reduction capacity during the ageing process.

## 1. Introduction

Beer flavour is determined by its chemical composition, which includes proteins and volatile flavour compounds such as esters, alcohols, fatty acids, sulphur compounds and ketones. The stability of these chemical components determines the shelf life of packaged beer. As the exportation of beer continues to grow, flavour stability has become an important issue for breweries. However, the ubiquitous nature of beer ageing has made it a vexing problem. Various flavour characters were developed in aged beers. The commonly accepted one of them is the cardboard stale flavour which is not present in fresh and vintage beers. Many factors are thought to be involved in beer ageing.

Both non-oxidative and oxidative processes can cause beer flavour deterioration [1]. Certain undesirable ethyl esters, heterocyclic compounds and carbonyl compounds are produced during fermentation without oxygen involved [1]. The carbonyl compounds, mainly aldehydes derived from Strecker degradation, are detrimental to beer flavour [2,3]. Recently, it has been recognised that the oxidative process plays a dominant role in beer flavour stability. Direct detection of reactive oxygen species (ROS) in aged beer using electron spin resonance spectroscopy has firmly implicated the role ROS in beer staling [4]. Hydroxyl radical [5], superoxide (O_2_ ^−^) [6] and hydrogen peroxide [7] are the key ROS in ageing process. The reason for the generation of beer ROS is most likely due to the unavoidable introduction of oxygen in the course of brewing and bottling. Molecular oxygen in wort or packaged beer can be reduced by accepting an electron to form superoxide (O_2_ ^−^), from which hydroperoxide (HOO^•^), hydrogen peroxide (H_2_O_2_), and the hydroxyl radical (HO^•^) can be derived [8]. Previous studies have demonstrated that these ROS react with beer components such as polyphenols, iso-α-acids and alcohols to produce off-flavor carbonyl compounds like aldehydes and ketones [9]. A particularly important aldehyde in the ageing process of beer is *trans*-2-nonenal, a product of lipid peroxidation, which gives rise to the cardboard or papery flavour distinctive to aged or stale beer.

The brewing industry have been attempting to maintain beer flavour stability and hence prolong its shelf life by minimising the oxygen content and hence ROS in the process of brewing using varieties of antioxidant compounds such as polyphenols, sulphites, sulphur dioxide and vitamins. However, little attention has been paid to one of the major component in beers–beer proteins. Many major antioxidants in living organisms such as bacteria, yeast and humans are rich in thiol activity. One of these is glutathione, a tripeptide, its thiol group is the key for its antioxidant activity [10]. This is also the case for the protein thioredoxin [11]. To determine whether thiol-containing polypeptides (proteins) in beer could also play a role in beer ageing by providing antioxidant activity we profiled the beer proteins in fresh, aged and stable vintage beers and isolated an interesting thiol-protein with antioxidant activity.

## 2. Results and Discussion

### 2.1. Sensory Analysis of the Fresh, Aged and Vintage Lager Beers

**Figure 1:**
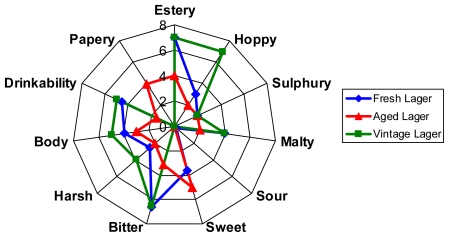
Spider web chart of the sensory analysis data. The flavours of the fresh, aged and vintage lager beer samples, assessed by an expert flavour assessment panel, were categorised into estery, hoppy, sulphury, malty, sour, sweet, bitter, harsh, body, drinkability and papery as described in the spider web chart.

Major constituents of beer are alcohols, vitamins, minerals, carbohydrates, proteins and flavour compounds [12]. The combined effect of these chemicals determines sensory properties of beer. In this study, the flavour panellists assessed the three kinds of beers at a time, namely the fresh, aged and vintage lager beers. The detailed scores of the fresh lager beer for each descriptor with statistical errors were estery (7 ± 0.5), hoppy (3 ± 0.2), sulphury (2 ± 0.2), malty (4 ± 0.0), sour (0.2 ± 0.1), sweet (3.6 ± 0.4), bitter (6.6 ± 0.5), harsh (2.5 ± 0.3), body (4 ± 0.5), drinkability (4.5 ± 0.6), and papery (0). The scores of the aged lager beer were estery (4 ± 0.2), hoppy (2 ± 0.2), sulphury (2 ± 0.2), malty (2 ± 0.3), sour (0), sweet (5 ± 0.5), bitter (3.1 ± 0.4), harsh (2 ± 0.2), body (3 ± 0.5), drinkability (1.5 ± 0.4), and papery (4 ± 0.8); and scores for the vintage lager beer were estery (7 ± 0.5), hoppy (7 ± 0.7), sulphury (2 ± 0.3), malty (4 ± 0.5), sour (0), sweet (0), bitter (6.5 ± 0.3), harsh (4 ± 0.5), body (5 ± 0.7), drinkability (5 ± 0.7), and papery (0). The data were illustrated by the spider web chart (Figure 1). These results demonstrated that the vintage beer had robust flavour stability as the fresh beer. Its tastes were far superior to the aged lager beer. In particular there was no evidence of *trans*-2-nonenal character that is the ‘papery’ character in the vintage beer. The aged beers got a papery character indicative of *trans*-2-nonenal; they were sweet and lost body and bitterness. The reason for the presence of off-flavour compounds in the aged beers during storage could be due to the oxidative process, whilst the redox balance of beer chemicals in the fresh and vintage beers may account for the beer flavour stability. As glutathione and other thiol molecules such as cysteine and protein sulfhydryls play significant antioxidant roles in non-beer systems like living organisms [13], beer thiol proteins could be key players in maintaining flavour stability. Thus, in order to delineate the sensory differences between these beers we characterised their thiol profiles to determine whether the thiol characteristic has changed among them.

### 2.2. The Level of Free Thiol Groups in Aged Beer was Low Compared to Fresh and Vintage Beer

**Figure 2:**
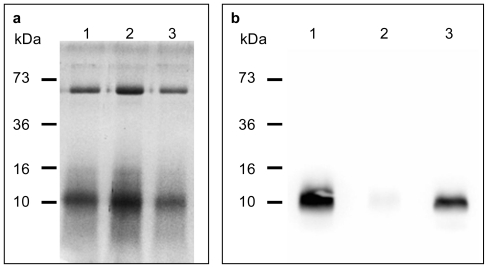
Characterisation of thiol-proteins in the beer samples by thiol labelling. (**a**) Fractionation of beer proteins in SDS-PAGE. Beer proteins from fresh lager beer (LB) (lane 1), aged lager beer (ALB) (lane 2) and 5-year-old vintage lager beer (VLB) (lane 3) were fractionated in 15% reduced SDS-PAGE and stained with Coommassie Blue dye. Protein bands were visualised by destaining the gel in 20% methanol and 10% acetic acid; (**b**) Thiol proteins in fresh LB, ALB and VLB. Beer proteins from fresh LB (lane 1), ALB (lane 2) and 5-year-old VLB (lane 3) were labelled with MPB and fractionated in 15% reduced SDS-PAGE. Protein bands were then transferred onto polyvinylidene diflouride (PVDF) membrane and probed with horse radish peroxidise-conjugated avidin. Labelled thiol proteins were visualised by enhanced chemiluminescence reagents (Amersham Biosciences, NJ, USA).

Free thiol-containing proteins in each of the beers were visualised using the free-thiol labelling reagent, MPB (*N*′-(3-maleidylpropionyl)) biotin and SDS-PAGE separation. Initial Coomassie Blue staining showed that each sample of beer contained 2 major protein bands at 43 kDa and 10 kDa and they were in similar concentrations (Figure 2a). Free thiol labelling revealed that only the 10 kDa protein contained free thiol groups. However, the intensity of this protein band was much higher in the lager beer (LB) and vintage lager beer (VLB) compared to the ‘aged’ ALB sample (Figure 2b). Considering the constant abundance of the 10 kDa protein in all 3 samples (Figure 2a) the results demonstrated that the disappearance of thiol groups of this protein in the aged beer was due to its oxidation. This finding, for the first time, showed that the 10 kDa beer thiol protein is strongly associated with beer flavour stability.

Beer proteins have not been the focus of research in the past years although prima facie evidences point to beer with higher protein content tends to be more stable [14]. As described by Bamforth [12], protein is the second highest component after carbohydrate. It is therefore very likely that beer proteins would contribute to sensory quality and flavour stability. In accordance to (Figure 2a), only two major proteins are present in beer, namely 43 kDa and 10 kDa proteins. This was in line with the other published studies [15]. There might be other beer proteins which are too low in quantity to be detected. To further characterise the 10 kDa and 43 kDa proteins, samples of the fresh LB beer were analysed by SDS-PAGE under non-reducing and reducing conditions.

### 2.3. Characterisation of the Secondary Structure of Beer Proteins under Non-Reducing and Reducing Conditions

**Figure 3:**
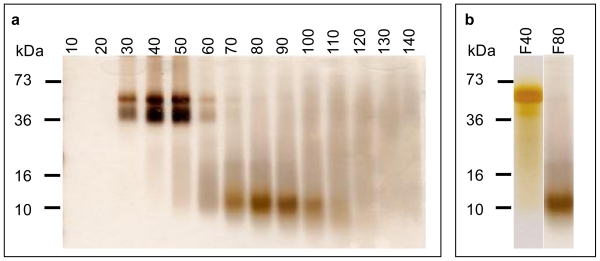

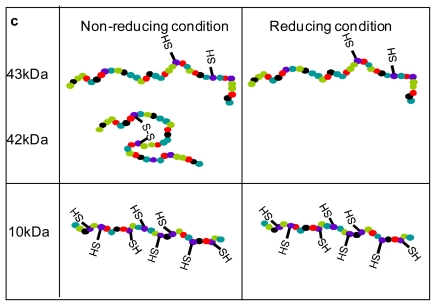
Characterisation of beer proteins: (**a**) Protein profile in non-reduced SDS-PAGE. The concentrated beer proteins were fractionated with a size-exclusion column. The protein profiles in every 10^th^ fraction of 300 fractions were resolved by 15% non-reduced SDS-PAGE and visualised by silver staining (3A). The fractions from 150^th^ onward were not shown due to lack of visible protein band; (**b**) Protein profile in reduced SDS-PAGE. Fractions 41 and 80 were fractionated in reduced SDS-PAGE and stained with silver nitrate in the same way as in (**a**); (**c**) Schematic illustration of intrapeptide disulphide bond formation. Proposed disulphide bonds or free thiols in the 43 kDa protein (fraction F40) and the 10 kDa protein (fraction F80) under non-reducing and reducing conditions.

Samples of the fresh LB beer were concentrated and proteins separated using Sephadex G50 chromatography. Three hundred fractions were collected and resolved by unreduced SDS-PAGE. Under non-reducing condition the 10 kDa was still run as a single protein band, however, the 43 kDa protein identified under reducing conditions was now fractionated as a doublet at around 42 kDa and 43 kDa (Figure 3a). These three major proteins were evident in the first 110 samples (Figure 3a) and no visible protein band was observed in Fractions 141 to 300 (results not shown). To analyse the effect of reducing conditions on the secondary structure of these proteins a reduced 15% SDS-PAGE was run and proteins were visualised by silver staining. The reducing condition made no difference to the single band pattern of the 10 kDa protein (Figure 3b) indicating that the protein is linearised in the beer samples. However, in the reduced condition the 43 kDa and 42 kDa proteins again migrated as one band, indicating that this protein has an alternate structure in beer samples due to different intra-disulphide bond formation as illustrated in (Figure 3c).

### 2.4. The 10 kDa Thiol Protein was Identified as the Barley Lipid Transfer Protein (LTP1)

**Figure 4:**
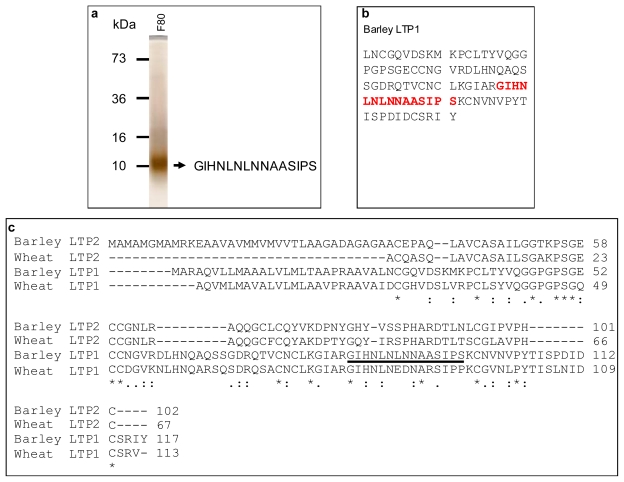
Identification of the 10 kDa beer thiol-protein by ESI-MS/MS peptide sequencing: (**a**) Peptide sequencing of the 10 kDa protein. Fraction 80 was run in 15% reduced SDS-PAGE. The 10 kDa bands were excised, trypsinised and sequenced by ESI-MS/MS; (**b**) Database matching of the sequenced peptide. The protein was identified as barley LTP1 by matching the sequenced peptide with protein databases (Swiss-Pro/TrEMBL, NCBI). The peptide (in red) in barley LTP1 is completely matched to the peptide in the 10 kDa beer protein; (**c**) Multiple sequence alignments. Multiple sequence alignments were performed with ClustalW2. “*” denotes identical amino acids. “:” and “.” denote conserved and semi-conserved amino acids, respectively. The sequenced beer LTP1 was underlined. The barley LTP1 (P07597), LTP2 (P20145) and wheat LTP1 (P24296), LTP2 (P82900) were retraced from Swiss-Pro/TrEMBL.

The 10 kDa protein was abundant in LB and VLB fresh quality beers and rich in free thiols, in order to understand its structure and function the protein was identified through peptide sequencing. The 10 kDa protein was first purified using protein chromatography and then further fractionated in a reducing SDS-PAGE gel. The protein band of 10 kDa was excised and trypsinised prior to mass spectrometry sequencing. As shown in (Figure 4a), a peptide, GIHNLNLNNAASIPS, was obtained and matched to barley lipid transfer protein (LTP1) in Swiss-Prot/TrEMBL protein databases (Figure 4b). Thus, the 10 kDa protein was identified as barley LTP1. Its mature peptide is 91 amino acids in length and contains 8 cysteine residues, nearly 9% of the whole protein (Figure 4b), demonstrating that it is indeed a thiol-rich protein. The complete amino sequence of LTP1 (P07597) was retraced from Swiss-Prot/TrEMBL protein database and aligned with its close relatives in Barley and wheat (Figure 4c). The homology between the two barley LTP isoforms was 24% while barley LTP1 was highly related to wheat LTP1 with 74% homology. The first 26 amino acids before leucine in barley LTP1 (Figure 4c) probably acts as a signal peptide for its transportation and secretion prior to being cleaved off. In terms of 43 kDa protein, it was identified as barly serpin Z7. The fact that only the 10 kDa LTP1 in fresh and vintage LB was positively labelled by free thiol-labelling reagent MPB suggests that beer LTP1 is rich in free thiols.

Considering the identification of 10 kDa and 43 kDa proteins as barley LTP1 and barley serpin Z7 respectively, these results also demonstrated that beer proteins are mainly originated from barley malt. According to their solubility in water, barley malt proteins are separated into three classes, namely water-soluble, water-insoluble and starch granule-associated proteins. Like any other cereal grains such as wheat and rye, about 50% of total protein is water-insoluble storage proteins such as high molecular weight glutenin subunits, low molecular weight glutenin subunits and hordein [16]. These proteins are mostly precipitated out during wort preparation. Major proteins pertinent to beer brewing are in water-soluble fraction such as amylases, which account for 20% of total grain proteins [17]. The numbers of proteins, which survive through multiple steps of malting, brewing and fermentation in the process of beer production, are small [18]. Until now, most of the studies on these beer proteins are related to the formation and stability of beer foams [17]. Beer LTP1 is found to be significant in foam formation. Interestingly, native barley LTP1 has no foaming property [19]. In the context of our finding that beer LTP1 is rich in free thiols, we think that free thiols could be partly responsible for its foaming capacity. However, most importantly, this study explored the relationship between the beer thiol protein and beer sensory properties. The presence of thiol-rich LTP1 in fresh and vintage LB indicates that this protein has a prominent role in maintaining redox balance of beer. To determine if the free thiols in LTP1 protein were involved in antioxidant capacity such as free radical scavenging and antioxidant activities, we assessed the protein using DPPH assay and a *Saccharomyces cerevisiae*-based assay.

### 2.5. Free Radical Reduction and Antioxidant Activities of Barley LTP1

**Figure 5:**
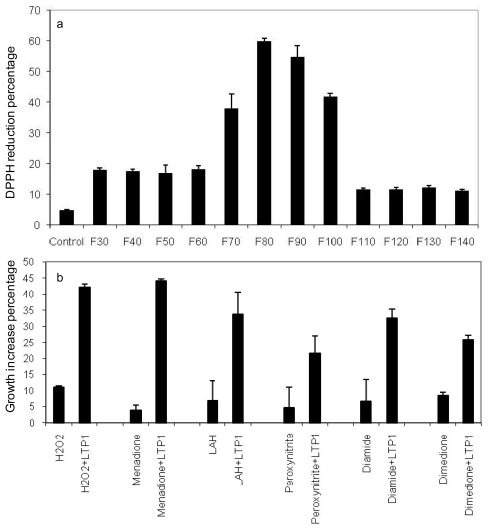
Free radical scavenging and antioxidant activities of the beer thiol-protein LTP1: (**a**) Free radical scavenging activity of LTP1. Protein fractions F30 to F140 were assay against DPPH (1,1-diphenyl-2-picrylhydrazyl). Scavenging activity was determined by percentage reduction of DPPH; (**b**) Antioxidant activity of LTP1. Antioxidant activities of the 10 kDa LTP1 in fraction F80 against 6 oxidants were measured using a *Saccharomyces cerevisiae* based assay. Addition of the 10 kDa protein at 2 mg/mL increased yeast growth by 31% against the buffer control at the end of 4 hr treatment under 4 mM H_2_O_2_.

Chromatographic fractions 80 and 90 of beer proteins in which LTP1 was present had significant free radical scavenging activity as a percentage of DPPH reduction (Figure 5a). In relation to fractions not containing LTP1, this activity of LTP1 was 3-fold higher. To further confirm LTP1’s free radical scavenging activity in a physiological context, it was tested against six reactive oxygen species using a *Saccharomyces cerevisiae*-based assay. The antioxidant activity of LTP1 protected the yeast against the toxic effects of all six oxidants (Figure 5b). LTP1 was most effective against menadione, increasing yeast growth by 11-fold, and it counteracted all the six oxidants and increased yeast growth approximately 4 or 5-fold against H_2_O_2_, linoleic acid hydroperoxide (LAH), peroxynitrite and diamide. These findings demonstrated barley LTP1 has free radical scavenging and antioxidant capacity.

Like other reducing antioxidants such as glutathione, the free thiols in the beer protein could be involved in elimination of free radicals and ROS as demonstrated by the data from DPPH reduction and yeast-based antioxidant assays, thus playing a role in flavour stability. Its strong free radical scavenging activity in DPPH assay correlates well with its antioxidant activity in the yeast-based assay. In terms of brewing, LTP1’s activity against hydrogen peroxide and LAH is significant. H_2_O_2_ and LAH are thought to major ROS involved in flavour deterioration process [7]. Elimination of these ROS abrogates the cause of oxidative process. Linoleic acid is found to be the most abundant lipid derived from malt and its oxidation by hydrogen peroxide or hydroxyl radical can lead to formation of LAH which can in turn trigger oxidative reactions, resulting in generation of precursors of the stale tasting aldehydes. The antioxidant activities of barley LTP1 as shown in (Figure 5b) may account for the flavour stability in fresh and vintage beer, while absence of its free thiols contributed to stale flavour in the aged beer.

The identification of the protein as barley LTP1 and its molecular and structural information are helpful to understanding the underlying basis for its antioxidant role. Barley LTP1 is a thiol-rich protein, containing 8 cysteine residues (Figure 4b). Its mature polypeptide starts with leucine (L). The first 26 amino acids before leucine (Figure 4c) probably acts as signal peptide for its secretion and transportation prior to being cleaved off. In its native form such as in mature barley grain, these cysteines form 4 intra-molecule disulfide bridges, leading to its 3D structure–four α-helices forming a central and conical hydrophobic core [20]. This high content of thiol cysteines in the protein is the basis for its radical scavenging and antioxidant activities. However, native barley LTP1 would not have antioxidant activity because all its thiol groups are occupied in the formation of disulfide bonds. The same goes true in lacking foaming capacity for native barley LTP1 [19]. The labelling of LTP1 thiols in beer demonstrated that the disulfide bonds in the native LTP1 were disrupted and linearised, most likely due to denaturing steps of malting, wort boiling and brewing. Its single band pattern in SDS-PAGE under reducing and non-reducing conditions (Figure 3) clearly demonstrated that beer LTP1 was completely denatured with no secondary structure. In contrast, the extra band of 42 kDa in non-reducing condition indicated that the 43 kDa beer protein was partially folded, which reduced its availability of free thiols and in turn rendered the protein ineffective in free radical scavenging and antioxidant activities. These free thiols were maintained during brewing and in packaged beer by a variety of factors. One of them could be the glycation of glycine and lysine residues with sugars such as glucose and xylose via the Maillard reaction [21]. The foam stabilising property of LTP1 has also been attributed to its glycosylation [19]. This notion is supported by the protein sequencing result. In the course of peptide sequencing, the 10 kDa protein band was excised and trypsin-digested. Thus, any peptides on the C-terminal side of lysine and arginine would be released by proteolysis and sequenced if these lysine and arginine residues are not followed by proline. The fact that the only sequenced peptide was derived from C-terminus of arginine suggests the lysine residues were modified by glycation which protected these lysines from proteolysis by trypsin. Such modification can also result in steric hindrance, preventing reformation of the disulfide linkages in LTP1.

The antioxidant activity of LTP1 in DPPH and yeast-based screening assays indicates the protein could function as ROS scavenger during fermentation and in packaged beer. A possible working mechanism is proposed as below:

(1) $$\text{LTP}-\text{SH}+{\text{H}}_{2}{\text{O}}_{2}\to \text{LTP}-\text{SOH}+{\text{H}}_{2}\text{O}$$

(2) $$\text{LTP}-\text{SOH}+\text{RSH}\to \text{LTP}-\text{SSR}+{\text{H}}_{2}\text{O}$$

(3) $$\text{LTP}-\text{SSR}+{{\text{SO}}_{3}}^{=}\to \text{LTP}-\text{SH}+{{\text{RS}-\text{SO}}_{3}}^{=}$$

In this model, LTP thiol(s) is oxidised to the sulfenic acid by oxidants such as H_2_O_2_, which results in the destruction of a peroxide molecule in 1:1 stoichiometry. The free thiol can be recovered by two sequential reactions (reactions 2 and 3). The reaction 2 generates a disulfide (LTP-SSR) through reaction with a small molecule (HS-R) such as yeast thioredoxin. The reaction 3 uses sulfite or phenolic compounds to generate free thiol from the disulfide for the next round elimination of ROS. There was abundance of small molecular compounds in beer which could be involved in these reaction cycles. The reductive compound-sulfite was normally present in beer, it can drive reaction 3. This model provides an important basis for brewers in practical manipulation for beer flavour maintenance. The findings of this study could also lead to a barley breeding program for producing a LTP1-enriched variety. In addition to all our findings, the high lysine content of 4.5% of the whole LTP1 protein (Figure 4b) offers an extra health benefit.

## 3. Experimental Section

### 3.1. Preparation of Fresh, Aged and Vintage Beers

The fresh, aged and stable vintage beers were commercial full strength Australian lager beers bottled in 375 mL crown sealed bottles. Fresh lager beer (LB) was straight from production line at Carlton and United Brewery, Foster’s Group, Australia and kept at 4 °C. Aged lager beer (ALB) was prepared by incubating LB at 30 °C for 12 weeks. Stable vintage lager beer, VLB, was bottled in 2003 and kept at 20 °C until 2007. Vintage beer was brewed once a year to coincide with the hop harvest in southern Australia. It could only be brewed as a one off speciality product because the recipe depends on the input of green hops. These were un-kilned hops that were picked and added almost immediately to the kettle boil of the wort prior to fermentation.

### 3.2. Sensory Analysis of the Beer Flavours

The flavour characters of fresh, aged and vintage lager beer samples were analysed by an expert flavour assessment panel using the sensory analysis technique called quantitative descriptive analysis (QDA) [22,23]. The expert sensory panel members, numbering 6–10, were highly trained to recognise individual flavour notes contributing to beer flavour. The flavour descriptors are derived from and represent most of the major items of the flavour wheel [24]. Panellists assess each beer in turn (up to 4 beers at a sitting) and score each for the appropriate flavour descriptors. The flavours were assessed with a graduated number score with 10 being a strong sense and 1 a low sense. Panellists’ flavour descriptors were estery, hoppy, sulphury, malty, sour, sweet, bitter, harsh, body, drinkability and papery.

### 3.3. Labelling of Beer Protein Thiols

The free-thiol labelling reagent, *N*′-3-maleimido-propionyl biocytin (MPB), was employed to probe protein thiols as previously described with modifications [25]. Beer proteins in fresh LB, ALB and VLB were concentrated by ultrafiltration, 100 μg protein of each beer sample was mixed in a 4:1 ratio with 0.2 M sodium phosphate, pH 8.0, resulting in a final volume of 0.5 mL. MPB was added to a final concentration of 0.5 mM and allowed to react for 30 min before un-reacted reagent was quenched by the addition of 2 mM dithiothreitol (DTT). Proteins in the samples were then fractionated by 15% sodium dodecyl sulphate polyacrylamide gel electrophoresis (SDS-PAGE), and thiol-labelled proteins were visualised after transfer onto a polyvinylidene diflouride (PVDF) membrane by reacting with avidin-peroxidase and enhanced chemiluminescence detection. A replicate polyacrylamide gel was not transferred but stained with Coomassie Brilliant Blue G-250 for validation of protein loading for each sample.

### 3.4. Beer Protein Purification

To purify beer proteins, fresh beer was first concentrated from 250 mL to 30 mL by ultrafiltration using a 5 kDa molecular weight cut-off membrane. The concentrated beer protein solution was spun at 13,000 rpm for 10 min in Eppendorf tubes to obtain clear protein supernatant. The protein concentration was measured using a bicinchoninic acid (BCA) quantification kit (Sigma, USA).

Size-exclusion protein chromatography was carried out using Sephadex G50 (5 × 10^3^ – 7 × 10^4^ Dalton, Amersham Biosciences, USA). Prior to loading sample, the column (60 cm in length, 2.5 cm in diameter) was packed and equilibrated with 10 bed-volumes (1.5 litres) running buffer (25 mM Tris/HCI, pH 7.4, 0.3 M NaCI). Fifty milligrams of beer protein extract in 6 mL was loaded onto the Sephadex column. The column was run under the following conditions: flow rate 1 mL/min (Wiz pump, ISCO, USA), UV detector 280 nm and sensitivity 2.0 (CIA-5, ISCO, USA), chart speed 1.5 cm/hr. Three hundred fractions in total (2 mL per tube) were collected using an automatic collector (LKB Bromma 2211 Superrac, Sweden) after 3 hr of running.

### 3.5. SDS-PAGE

The chromatographic fractions were analysed by 15% polyacrylamide non-reducing SDS-PAGE gel with 29:1 of acrylamide:bisacrylamide ratio [26]. Each sample (10 μL) was mixed with an equal volume of Laemmli SDS-sample buffer (62.5 mM Tris-HCI pH 6.8, 2.0% SDS, 10% glycerol, 0.005% bromophenol blue) and boiled for 5 min using a digital dry bath (Labnet, USA), and then cooled on ice. The 15% separation or resolving gel was prepared with a acrylamide:bisacrylamide solution at a ratio of 29:1 in 250 mM Tris-HCI, pH 8.8, 5% glycerol, and 5% stacking gel in 189 mM Tris-HCI, pH 8.8. Both gel components contained 0.1% SDS. The wells were rinsed with Tricine running buffer (25 mM Tricine, 400 mM glycine, 0.1% SDS) using a syringe with a 24G 1 inch needle (Terumo, Tokyo, Japan) before loading. Electrophoresis was carried out at 30 V per gel for 3 h until the dye had run to the edge of the gel. Protein profile was visualized by silver staining [27].

### 3.6. Free Radical DPPH Reduction Assay

The protein fractions were assessed for antioxidant activity using the DPPH assay established by Blois [28]. The assay was done in a 96-well microtitre plate format. The antioxidant, ascorbate (Vitamin C), was used as a positive control. A standard curve was included for each plate with a series of ascorbate concentrations (0, 10, 20, 40, 60, 80, 100, 200, 400 and 1000 μM). Samples, 2.0 μg each of 50 μL volume, were added to wells and the assay was started by adding 150 μL of 62.5 μM DPPH. After 10 min incubation, reactions were quantified by measuring the absorbance at 517 nm using a microplate reader (Multiskan EX, Thermo Electron, USA). The percentage of DPPH reduction for each fraction was calculated against the ascorbate standard curve.

### 3.7. Saccharomyces Cerevisiae-Based Antioxidant Assay

The assay was conducted according to Wu *et al*. [29]. *Saccharomyces cerevisiae* BY4743 was cultured overnight in a 30 mL volume by inoculation of a single colony. The culture was then diluted to an optical density at 600 nm (OD_600_) of 0.2 in media. Concentration for each oxidant that resulted in growth arrest for a length of 2–4 hr was first determined. These arresting concentrations were 4 mM for hydrogen peroxide, 75 μM for LAH, 150 μM for menadione, 10 mM for peroxynitrite 5 mM for diamide and 10 mM for dimedione. The antioxidant activity of the beer protein was measured on the basis of its capacity to restore yeast growth from oxidant-induced arrest by reducing the inhibitory effect of oxidants. The diluted yeast was mixed with 2.0 μg LTP1 contained in 50 μL fraction 80 and individual oxidants: H_2_O_2_, cumene hydroperoxide (CHP, an aromatic hydroperoxide), linoleic acid 13-hydroperoxide (LAH, a product of lipid peroxidation), menadione (superoxide-generating agent), peroxynitrite and the thiol oxidants-diamide and dimedone. Yeast solution was aliquoted 150 μL into each of six replicate wells. The plates were incubated in 30 °C warm room with shaking at 1000 rpm. Yeast growth was monitored by reading OD_600_ using a microplate reader (Multiskan EX, Thermo Electron, USA). Oxidant-only controls and no-oxidant controls were included in the experiment.

### 3.8. Peptide Sequencing by ESI-MS/MS

The purified beer protein in Fraction 80 was sequenced with mass spectrometry as follows. The protein band of interest was excised and cut into tiny pieces and washed in 40% acetonitrile (v/v), 50 mM ammonium bicarbonate, pH 7.8, then dried under vacuum centrifugation for 25 min and rehydrated at 4 °C in 15 μL of sequencing grade trypsin solution (15 ng/μL trypsin in 50 mM ammonium bicarbonate, pH 7.8) for 1 h. The sample was then digested overnight in trypsin solution at 37 °C. Peptide mixtures were extracted in 10% (v/v) acetonitrile and 1% (v/v) TFA. For peptide sequencing using ESI-MS/MS, peptide mixtures were concentrated and desalted using custom-made chromatographic micro-columns. Poros reverse-phase (R2) material (20 μm bead size, Applied Biosystems, USA) was packed in a constricted GELoader tip (Eppendorf, Hamburg, Germany). A 10 mL syringe was used to force liquid through the tip-column by applying gentle pressure. The tip-columns were equilibrated with 10 μL 1% formic acid. The peptide mixture was loaded onto the column. Bound peptides were washed with 20 μL 1% formic acid and eluted into a borosilicate nano-electrospray capillary (Micromass, UK) using 70% acetonitrile/1% formic acid. ESI-MS/MS was carried out using electrospray time-of-flight mass spectrometry (LC/MS/TOF) (Q-Star Pulsar I, Applied Biosystems, USA). The nano-electrospray needle containing sample was mounted in the source and stable flow was obtained by capillary voltages of 900–1200 V. Precursor ion scans were performed to detect m/z values for peptides. The m/z of individual precursor ions were selected for fragmentation using collision energies of 18–30 eV with the collision gas (argon). Fragment ions were processed by MassLynx Version 3.4 (Micromass, UK). Peptide sequences were deduced by the mass differences between y- or b-ion ‘ladder’ series using the Mascot sequence matching software (Matrix Science, USA) and confirmed by manual interpretation. Peptide sequences obtained were matched with the NCBInr database, Viridaeplantae taxonomy using the protein BLAST search program (http://www.ncbi.nih.gov/BLAST). Complete amino acid sequence of the identified protein was obtained from Swiss-Prot/TrEMBL protein database through ExPASy server (Expert Protein Analysis System). Multiple sequence alignment was performed with ClustalW2.

## 4. Conclusions

A systematic protein analysis, including SDS-PAGE, free thiol labelling, peptide sequencing and database alignment, was carried out with fresh, aged and vintage beer samples. The results demonstrated, for the first time, that a 10 kDa free thiol protein was abundantly present and completely denatured in fresh and vintage beers. The protein was purified and identified as barley LTP1. The free radical scavenging and antioxidant assays further revealed that LTP1 in fresh and vintage beers had significant free radical scavenging and antioxidant activities. Taken together these findings with the sensory assessment data, we conclude that the thiol groups in LTP1 play an important role in the protein’s antioxidant functionality and beer flavour stability.
